# Offset or not: guidance on accounting for sampling effort when modelling abundance data

**DOI:** 10.1007/s00442-026-05944-z

**Published:** 2026-07-30

**Authors:** James A. Smith

**Affiliations:** 1 NSW Department of Primary Industries and Regional Development, Port Stephens Fisheries Institute, Locked Bag 1, Nelson Bay, Sydney, 2315 New South Wales Australia; 2 School of Biological, Earth and Environmental Sciences, University of New South Wales, Sydney, New South Wales 2052 Australia

**Keywords:** GLM, Survey effort, Catch standardisation, Regression, Species distribution modelling

## Abstract

Ecological data are often dependent on sampling effort, such as counts measured per unit area. Such data come from monitoring programs across ecology and fisheries (e.g. point counts, camera and trap surveys, fishery sampling), and underpin abundance indices, species distribution models, and often management decisions. A common tool to account for effort differences is the ‘offset term’ in generalized linear models, enforcing fixed proportionality between effort and the response. However, detailed guidance is limited on applying offsets and transformations, or when an effort covariate is preferable. This article reviews approaches for modelling sampling effort in regression analyses, provides best-practice recommendations, and uses simulation to examine performance of alternative parameterisations across scenarios. Offsets of log-transformed effort are preferable when proportionality is strongly supported, guaranteeing proportionality and avoiding misspecification bias. When deviation from proportionality is possible (which may be common), effort should be included as a log-transformed covariate, ideally a constrained smoother, to allow estimation of non-linear or saturated relationships. The choice between offset and covariate also depends on modelling aims: offsets support standardisation, whereas covariates enable inference on effort effects. In delta or hurdle models, effort covariates are useful because offsets have different interpretations across model components. Additional considerations include multiple effort variables, collinearity, endogeneity (when effort responds to abundance), and species-specific responses in multi-species models. Rather than modelling effort routinely, researchers should explore the effort–response relationship (proportional, otherwise linear on the link or original scales, or non-linear), drawing on prior knowledge, practical experience, data exploration, and statistical tests.

**Supplementary Information:**

The online version contains supplementary material available at 10.1007/s00442-026-05944-z.

## Introduction

Survey programs and related data often have variation in sampling effort, such as differences in hours searched or areas surveyed. Accurately accounting for this variation is crucial when modelling response variables like abundance (e.g., counts or biomass) to ensure unbiased estimates of both the response and other predictor variables (e.g., environmental or spatiotemporal covariates). One common approach is to transform the response variable before modelling, such as calculating counts per unit of sampling effort. However, this approach can introduce statistical issues, such as violating the assumptions of count or biomass data distributions or misrepresenting the variance structure (Zuur et al. 2009).

An alternative and often preferred method is to use a generalized linear model (GLM), or similarly a generalised additive model (GAM), that includes sampling effort as a transformed offset term or covariate (Maunder and Punt 2004; Zuur et al. 2009). This approach models the response variable on its original scale while standardizing it to sampling effort. Effort, meaning here the recorded extent of a sampling event (e.g. duration, distance, area, or number of units deployed), influences the observation process rather than the underlying ecological state. Effort sits alongside other observation-level variables such as observer skill, gear characteristics, or time-of-day (Bas et al. 2008; Johnston et al. 2018) – all variously termed ‘detectability covariates’ (Buckland et al. 2009; Thorson and Kristensen 2024) or ‘catchability covariates’ (Thorson 2019).

Many studies have included effort as an offset, for effort variables such as the number of trap nights (Kammerle et al. 2018), distance walked (O’Kelly et al. 2018), number of survey points (Ausprey et al. 2023), and area trawled (Thorson et al. 2020). Recent good-practice guidance for fishery catch rate modelling identifies the offset as an option but does not detail when it is appropriate (Hoyle et al. 2024). There remain numerous decisions to make, and pitfalls to avoid, when including sampling effort in a GLM. These include understanding the assumed relationship between effort and the outcome variable, and the extent to which a fitted covariate can model potential relationships.

A variable included as an offset is used to adjust the expected value of the response without having an estimated parameter, in contrast to a covariate, whose effect is instead estimated from the data. This means the offset variable has a fixed coefficient value = 1, an assumption not required for a covariate. A sampling effort offset is typically only used when a GLM uses a log link function, which encompasses the common statistical distributions for abundance data: Poisson, negative binomial, and Tweedie, but also lognormal and gamma in delta (hurdle) models (Zuur et al. 2009; Thorson 2018). Because the offset term is used to standardize the response (i.e. abundance per unit sampling effort), the response and effort variables need to be on the same scale, which means log-transforming effort to match the link function. In other words, the log link function allows the offset to scale the expected value of the response in a proportional way on the original scale, i.e. a 50% increase in effort means a 50% increase in abundance, all else being equal.

A general log link GLM, with an effort term *T*, can be written:

1 $$\:{Y}\sim{F}\left(\mu\:,\theta\:\right),\:\mathrm{E}\left(Y\right)=\mu\:$$

2 $$\:\mathrm{log}\left(\mu\:\right)={\beta\:}_{0}+{\beta\:}_{1}{X}_{1}+\:{\beta\:}_{2}\mathrm{log}\left(T\right)$$

3 $$\:\mu\:={e}^{\left({\beta\:}_{0}+{\beta\:}_{1}{X}_{1}\right)}\times\:{T}^{{\beta\:}_{2}}$$

The response variable *Y* is a random variable with statistical distribution $\:{Y}\sim{F}\left(\mu\:,\theta\:\right)$, and the log link function determines the relationship between the expected value of abundance $\:\mathrm{E}\left(Y\right)=\mu\:$ and the predictor variables (the linear predictor). *β*_0_ is the intercept, *β*_1_ is the coefficient (estimate) for the first predictor variable *X*_1_, and *β*_2_ is the coefficient of the sampling effort term *T*. GLMs model the expected value of *Y*, not *Y* itself, and the GLM can be written equivalently on the link scale (Eq. 1) or the original ‘response’ scale (Eq. 2). Equation 2 shows that when log-transformed *T* is included in the GLM as an offset term, *β*_2_ = 1 (the parameter is not estimated), so effort is proportional to expected abundance, i.e. we are essentially modelling $\:\mu\:/T$; this is the same as modelling abundance as a rate (e.g. number per hour).

When modelling abundance (including outcome variables such as fishing catches) the options to (1) include sampling effort as an offset or (2) include it as a covariate, are often presented as equally able alternatives (Maunder and Punt 2004; Thorson 2019). The second option is often considered more flexible than an offset term – capable of fitting a proportional relationship as well as deviations from it – with deviations possible due to processes like the saturation of fishing gear (Groeneveld et al. 2003; Bacheler et al. 2013; Kuriyama et al. 2019; Hanamseth et al. 2022), predation of captured animals (Faure et al. 2025), or trophy hunting (Allen et al. 2020). Non-linearity of the effort–abundance relationship may not be uncommon, especially for ‘capture’ sampling methods (Thorson 2019; Smith et al. 2020; Smith and Johnson 2024).

For this flexibility to be true, the effort variable included as a covariate must also be able to represent the proportional effort–abundance relationship implied by an offset (i.e. *β*_2_ can be = 1 in Eq. 2). This depends on model structure and likely on the collinearity among covariates – when covariates are collinear, parameter estimates can become unstable, have inflated uncertainty, and their effects difficult to separate (Dormann et al. 2013). In this case, if effort is correlated with variables such as season or temperature, its estimated slope may be unreliable and confounded with those variables. One obvious pitfall when modelling effort is not log-transforming effort *T* before inclusion as a covariate:

$$\:\mathrm{log}\left(\mu\:\right)={\beta\:}_{0}+{\beta\:}_{1}{X}_{1}+{\beta\:}_{2}T$$

$$\:\mu\:={e}^{\left({\beta\:}_{0}+{\beta\:}_{1}{X}_{1}+{\beta\:}_{2}T\right)}$$

**Fig. 1:**
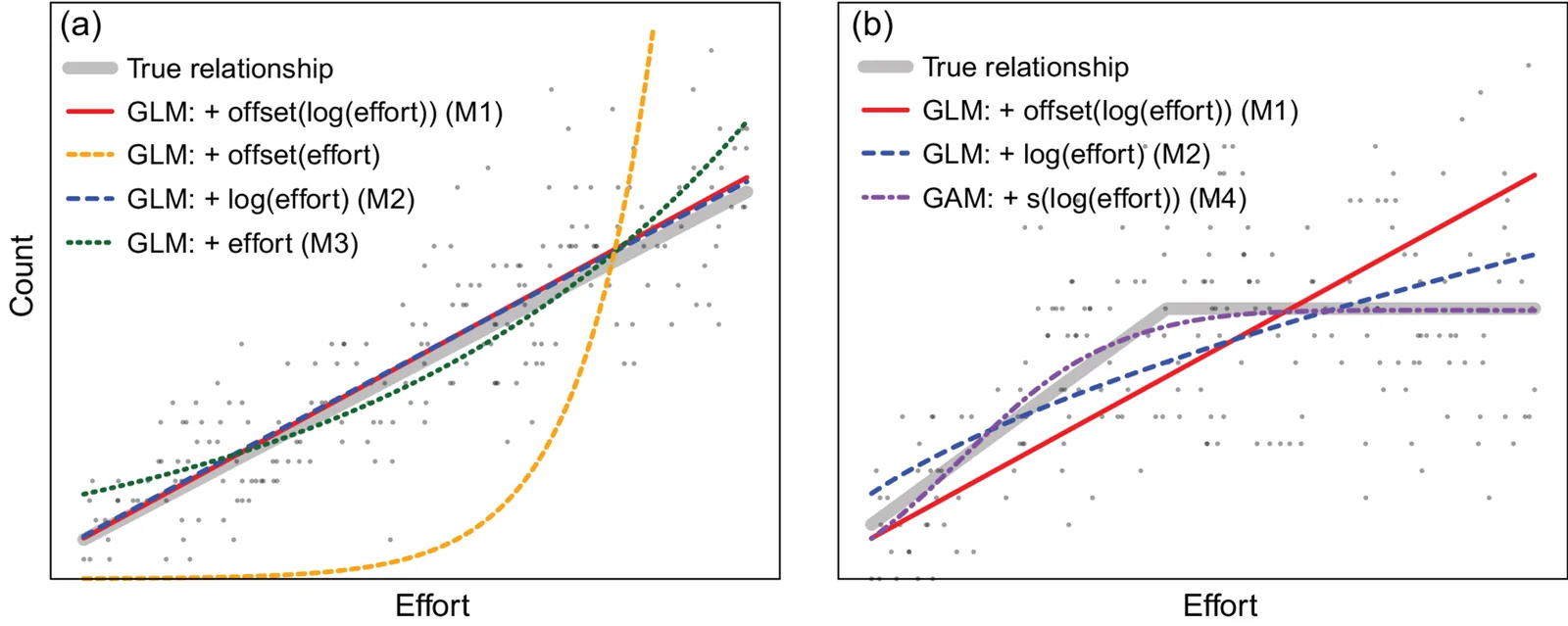
Example effort-abundance relationships and various effort parameterizations, using synthetic data (*N* = 200). This shows how different effort parameterisations can fit a (**a**) proportional or (**b**) thresholding effort–abundance relationship for simulated count data (a generalized additive model, GAM, is considered a special cased of GLM). In this simple case, the fit is poor when effort is not log transformed (**a**), and only a smoother can accurately estimate this thresholding relationship (**b**), although the + log(effort) option can be non-linear. The model number (e.g. M1) corresponds to the model detailed in Table 1. Code for generating this figure is shared in the GitHub repository

In this case, effort is no longer proportional to expected abundance and the relationship between effort and abundance is non-linear, even if *β*_2_ = 1 (Fig. 1a). This model is only wrong if the user was assuming the model could act like an offset term and fit a proportional effort–abundance relationship if it existed, but would create an unlikely effort-abundance relationship.

**Table 1:** Summary of the eight data scenarios (D1 to D8). Collinearity was induced with a covariance of 0.5 (effort–temperature) or -0.5 (effort–site). The threshold effort–abundance relationship was proportional up until the mean effort value (12 units) and then constant at larger effort values. A supplementary analysis testing other covariance values is shown in Fig. S3

No.	Effort–abundance relationship	Collinearity of predictors	Temperature effect
D1	Proportional	None	Linear
D2	Proportional	Effort–temperature	Linear
D3	Threshold	None	Linear
D4	Threshold	Effort–temperature	Linear
D5	Proportional	Effort–temperature	Domed
D6	Proportional	Effort–temperature and effort–site	Linear
D7	None	None	Linear
D8	Threshold	Effort–temperature and effort–site	Domed

The goal of this article is to provide guidance on the appropriate model structures for accounting for sampling effort in GLMs, helping researchers avoid common pitfalls and understand the implications of different modelling choices. This is achieved by generating data with different effort-abundance relationships and levels of collinearity among covariates, and by evaluating which approach for including sampling effort in a model is most robust to these different relationships. Given that the effort–abundance relationship is not usually known, a specific aim is to identify whether there is one model structure that is generally robust for modelling an unknown effort–abundance relationship that could be either proportional or non-linear. I end by highlighting some issues that are easily overlooked when accounting for the wide variety of sampling effort variables in statistical models.

## Demonstrating different model structures

### Data scenarios

Eight data scenarios (D1–D8; Table 2) and seven model structures (M1–M7; Table 1) are presented, of which six were tested (M1–M6), with R code (v4.5.0, R Core Team 2025) available at https://github.com/smithja16/Effort_Offset_Simulation. Abundance data consisted of 1200 counts from a negative binomial distribution (dispersion parameter, theta = 3.0), generated from specified relationships with three predictors: sampling effort (continuous, arbitrary units), site (categorical, 3 levels), and temperature (continuous). These specified relationships mean that model success can be measured by the recovery of known patterns. The negative binomial distribution was used because it is a widely used distribution for overdispersed ecological counts (Warton et al. 2016) and is a conservative (lower signal-to-noise) choice, and testing using a Poisson distribution showed the same general findings. Data scenarios varied across three factors: (1) the relationship between effort and abundance (none, proportional, threshold; where ‘threshold’ is proportional up to the mean effort value and constant at higher effort), (2) the collinearity among effort and the other predictor variables (collinear or not), and (3) the shape of the temperature effect (linear or domed [i.e. unimodal or bell-shaped] on the scale of the linear predictor). The eight data scenarios (D1–D8) are summarised in Table 2 and cover sufficient combinations of the three factors to evaluate their influence. The intent is to illustrate common considerations rather than provide an exhaustive treatment of potential scenarios.

**Table 2:** Syntax (using the R software language) and equations for common ways to specify effort in a GLM. All models have a log-link, except the last which has an identity link. For all models, abundance *Y* is a random variable with statistical distribution$\:Y\sim{F}\left(\mu\:,\theta\:\right)$, and the link function determines the relationship between the expected value of abundance$\:\mathrm{E}\left(Y\right)=\mu\:$and the predictor variable(s). *β*_0_ is the intercept, *β*_1_ is the estimate (coefficient) for the first predictor variable *X*, and *T* is sampling effort. s indicates a smoother of function *f*. For the final model, *c* is a constant (< min(*Y*/*T*) to avoid log(0), and *σ*^2^ is the variance of the residuals on the log scale. The offset function in R ensures that no parameter is estimated for that variable (Zuur et al. 2009)

No.	*R* syntax and Equation	Implied effort–abundance relationship
M1	glm(Y ~ X + offset(log(effort))) $\:\mathrm{log}\left(\mu\:\right)={\beta\:}_{0}+{\beta\:}_{1}X+\mathrm{log}\left(T\right)$ $\:\mu\:={e}^{\left({\beta\:}_{0}+{\beta\:}_{1}X\right)}\times\:T$	Abundance is proportional to effort on the original scale, i.e. 10% higher effort means 10% higher mean catch; this is the typical model structure for an effort offset; transforming in the model formula offset(log(effort)) or using a transformed effort variable offset(log_effort) are equivalent
M2	glm(Y ~ X + log(effort)) $\:\mathrm{log}\left(\mu\:\right)={\beta\:}_{0}+{\beta\:}_{1}X+{\beta\:}_{2}\mathrm{log}\left(T\right)$ $\:\mu\:={e}^{\left({\beta\:}_{0}+{\beta\:}_{1}X\right)}\times\:{T}^{{\beta\:}_{2}}$	Abundance is proportional to effort when *β*_2_ = 1 (as above), otherwise the relationship follows a power law, e.g. when *β*_2_ = 2 if effort doubles mean abundance increases by a factor of 4
M3	glm(Y ~ X + effort) $\:\mathrm{log}\left(\mu\:\right)={\beta\:}_{0}+{\beta\:}_{1}X+{\beta\:}_{2}T$ $\:\mu\:={e}^{\left({\beta\:}_{0}+{\beta\:}_{1}X+{\beta\:}_{2}T\right)}$	Abundance is non-linear with effort on the original scale; effort has a consistent multiplicative effect on the outcome, i.e. for every *unit* increase in effort abundance increases by a factor of exp(*β*_2_)
M4	gam(Y ~ X + s(log(effort))) $\:\mathrm{log}\left(\mu\:\right)={\beta\:}_{0}+{\beta\:}_{1}X+f\left(\mathrm{log}\left(T\right)\right)$ $\:\mu\:={e}^{\left({\beta\:}_{0}+{\beta\:}_{1}X+f\left(\mathrm{l}\mathrm{o}\mathrm{g}(T\right)\right)}$	The function *f* can create a variety of non-linear relationships, but when this is a straight line of slope = 1 it is equivalent to M1 with a proportional effort–abundance relationship
M5	gam(Y ~ X + s(effort)) $\:\mathrm{log}\left(\mu\:\right)={\beta\:}_{0}+{\beta\:}_{1}X+f\left(T\right)$ $\:\mu\:={e}^{\left({\beta\:}_{0}+{\beta\:}_{1}X+f(T\right))}$	This lies somewhere between M3 and M4 due to the flexibility of function *f*. But because most GAM smoothers *s* have the same degree of smoothness across a variable, the smoothness will change as *µ* changes, so this may not be capable of a fitting a perfectly proportional relationship (unlike M4)
M6	gam(Y ~ s(X) + s(log(effort))) $\:\mathrm{log}\left(\mu\:\right)={\beta\:}_{0}+f\left(X\right)+f\left(\mathrm{log}\left(T\right)\right)$ $\:\mu\:={e}^{\left({\beta\:}_{0}+f\left(X\right)+f\left(\mathrm{l}\mathrm{o}\mathrm{g}(T\right)\right)}$	Identical to M4 but with a smoother for *X* (temperature), so that the domed temperature effect of scenarios D5 and D8 could be fit properly; explores whether the effort covariate is less accurate due to misspecification of a collinear term (M4 vs. M6 for D5)
M7	glm(log(Y/effort + constant) ~ X, family=gaussian(link=”identity”)) $\:\mathrm{log}\left(\frac{Y}{T}+c\right)={\beta\:}_{0}+{\beta\:}_{1}X$ $\:Y=\left({e}^{\left({\beta\:}_{0}+{\beta\:}_{1}X+\frac{{\sigma\:}^{2}}{2}\right)}-c\right)\times\:T$	Equivalent to a linear model with log-transformed response, the relationship between abundance and effort is proportional by design; predictions of mean *Y* require bias-correcting the linear predictor with$\:+\frac{{\sigma\:}^{2}}{2}$, assuming that *Y* is log-normally distributed on the original scale (Duan 1983)

The effort–abundance relationship determines whether an offset term is appropriate, i.e. when it is ‘proportional’ an offset term would be expected to perform well (Hoyle et al. 2024), but when it is not a more flexible approach may be better. The collinearity between predictors evaluates whether certain model structures are more accurate when effort is correlated to other variables (e.g. more sampling occurs at certain sites or at specific temperatures), a pattern arising from preferential sampling (Diggle et al. 2010). It is possible that a collinear effort covariate may lead to less accurate recovery of a proportional effort–abundance relationship than an offset term; this is examined across a range of effort–temperature covariance (0–0.9) as a supplementary analysis. The shape of the temperature effect (linear or domed) evaluates whether misspecifying one covariate (e.g. fitting a domed effect with a linear term) influences the accuracy of the effort term, especially if the two terms are collinear (Dormann et al. 2013). Collinearity was induced using a multivariate normal distribution, and the domed temperature relationship was generated using a normal distribution.

## Model structures

Seven model structures (M1– M7) that differ primarily in how they model effort are shown in Table 1. Model 1 is the typical ‘effort as offset’ structure, and the others include effort as a covariate (Models 2–6), specifically as a smoother (Models 4–6) in a GAM to allow more flexibility. A smoother is a flexible shape estimated from the data and penalised to avoid overfitting (Wood 2017). Model 7 is a log-linear model that transforms the response before fitting, and was added for interest due to its occasional use (e.g. Shono 2008). For this analysis I tested models M1-M6, where I included two additional covariates, Site as a fixed factor and Temperature as a continuous variable. The log-linear M7 is structurally equivalent to M1 with respect to the effort–response relationship, so was not tested here. M6 was added to explore the misspecification data scenario described above, but it is identical to M4 in how it models effort. All models were fit using the ‘mgcv’ R package (v1.9-1, Wood 2017).

## Performance metrics

I evaluated success of the models across data scenarios by looking primarily at the (1) diagnostics of residuals, (2) model goodness of fit, and (3) parameter recovery and the effort marginal effect. Residuals (vs. fitted values and vs. the offset term) should be pattern free, indicating a well specified model. I used simulated residuals generated by the ‘DHARMa’ R package (v. 0.4.7, Hartig 2022). For goodness of fit I calculated the similarity of simulated and predicted abundance counts using repeated (*n* = 10) k-folds (k = 5) cross validation. Similarity was measured using the mean absolute error (MAE), and calculated for: (a) all data, (b) for data at large effort values, (c) for data at low effort values. MAE at large and small effort values tests whether an effort–abundance relationship fits well throughout the data. Differences in collinearity and the temperature effect among data scenarios cause differences in mean abundance, so MAE should only be compared among models within each data scenario.

AIC was also evaluated as a supplementary metric to highlight model parsimony, because it penalises additional parameters (e.g. effort as a covariate). However, AIC is an in-sample metric that assesses fit across the whole dataset, so cross-validation was used as the primary result because it captures out-of-sample performance and overfitting which could occur with more flexible parameterisations. Parameter recovery refers to the similarity in the specified and estimated values of the variable effects (i.e. coefficients or non-linear shapes) and dispersion, and a well specified model will have accurate parameter recovery. And the effort marginal effect refers to the shape of the estimated effort–abundance relationship (Fig. 1). Here, parameter recovery is judged successful when the 95% confidence interval of an estimate encompasses the true value used to generate the data, and likewise when the true effort–abundance relationship lies within the 95% confidence intervals of the estimated marginal effect.

## Model performance

### Residuals and dispersion

All models showed sufficiently pattern-free residuals, except for D7 (no effort effect) for the one model which used an offset and thus assumed a proportional relationship (M1; Fig. S1a). Not accurately modelling a threshold effort relationship (e.g. M1 for D8) did not significantly affect the residuals or dispersion (Fig. S1b-c).

### Goodness of fit

The model with a transformed offset term (M1) performed well when the effort–abundance relationship was proportional, because that is precisely what the offset assumes. The model that included effort as a transformed smoother (M4) performed well over both proportion and thresholding scenarios (Table 3; raw values Table S1), because it can represent a straight, slope-one (proportional) relationship but also bend to fit non-proportional shapes. Accuracy at the limits of the data (i.e. low and high effort values) was also generally better for the models with an effort smoother, even when tested against out-of-sample data. The inaccuracy from using an untransformed effort variable (M3) was largest at high and low effort values (Table 3). The AIC results support these patterns, and there is little cost (≤ 2 AIC points) to including effort as a covariate, even when the relationship is truly proportional (Table S2).

**Table 3:** Percentage difference in mean absolute error (MAE) among models. This compares the five models’ relative accuracy, determined using out-of-sample data from repeated cross validation. The percent difference is relative to the model with the lowest MAE in each data scenario, e.g. M1 had an MAE on average 0.08% higher than the lowest MAE over the four proportional data scenarios. M6 is not included in this comparison because it models temperature differently, and it otherwise identical to M4. The two models with the best performance for each metric are highlighted italic

	% MAE		% MAE high effort		% MAE low effort	
	Proportional	Threshold or constant	Proportional	Threshold or constant	Proportional	Threshold or constant
M1	*0.08*	3.13	1.20	22.16	*0.91*	0.92
M2	0.11	0.82	1.07	7.06	2.05	6.92
M3	0.74	1.40	4.39	8.28	9.77	16.09
M4	*0.03*	*0.02*	*0.29*	*0.30*	*1.50*	*0.86*
M5	0.11	*0.01*	*0.50*	*0.04*	2.21	*0.39*

**Fig. 2:**
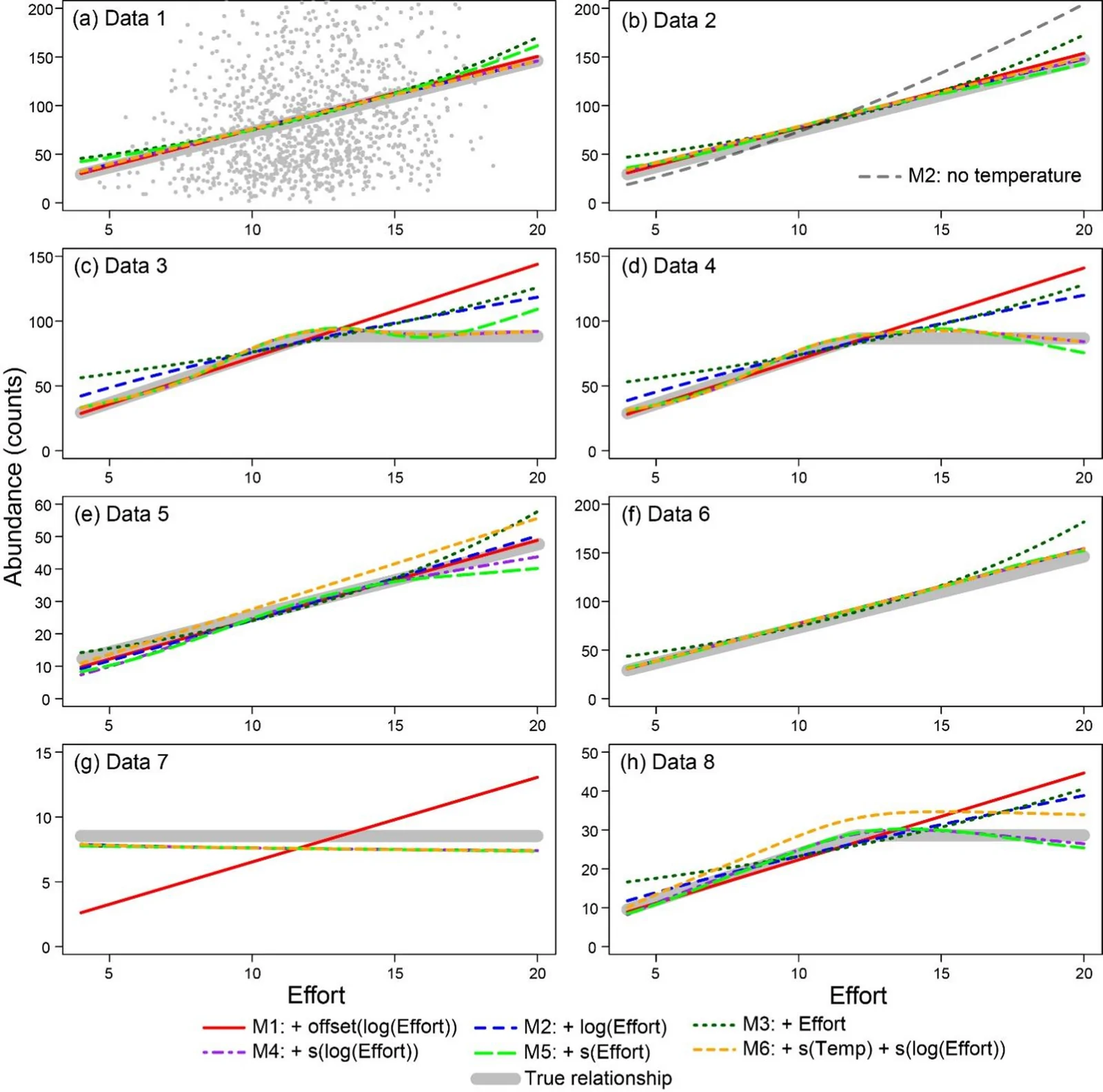
The true and estimated marginal effort–count relationships for (**a-h**) the eight data scenarios and six model structures. The true relationships used to generate the data are shown as thick grey lines (this marginal is approximate in any scenario with collinear variables). The generated data (*N* = 1200) are shown in **a**), highlighting the noise inherent in the negative binomial distribution as well as variation in the temperature and site effects. In **b**) is shown the effort marginal estimated by a version of M2 without the temperature covariate (grey dotted line); this illustrates that inducing collinearity between temperature and effort does influence the estimated effort effect

The difference in MAE among models was often small (Table 3), especially for proportional relationships, showing that most parameterizations fit the bulk of the data well (Fig. 2). This was somewhat a feature of the data generation, where there were fewer high and low effort values to fit. The difference in MAE among models is also a function of the unexplained information or noise; e.g. when a Poisson distribution is used for data generation instead of a negative binomial the percentage differences among models increases several fold due to the higher signal-to-noise ratio. The simulation results are expected to generalise beyond the negative binomial to all distributions commonly used for ecological count or biomass data, because they share a log link, so the linear predictor, and thus the effort parameterisation and its consequences, is identical across them. Delta (hurdle) models require careful attention (see Recommendations).

## Parameter recovery and Effort marginal effects

Parameter recovery was generally good (Table S3, Fig. S2), as seen in the true and estimated effort marginal effects (Fig. 2). All models accurately recovered the relevant temperature effects and site effects for all data scenarios. However, the intercept (representing reference site A) was poorly recovered by most models, especially when the model contained a smoother (M4-M6) or untransformed effort term (M3). The poor recovery would be typical of such data, due to the intercept representing a reference far from the simulated data (i.e. when effort or log(effort) and temperature = 0), and due to the compounded parameter uncertainty. Poor recovery was sometimes expected (e.g. a linear effort could not recover a thresholding relationship), but some cases were unexpected: M1 failed to recover the temperature effect when an offset was used to represent a thresholding effort–abundance relationship (D4), and M5 sometimes failed to recover the proportional effort relationship (D1, D5; Fig. 2).

## Implications of simulation results for offsets and covariates

The data scenarios and model structures allowed me to evaluate five questions:

1. *Which models failed to fit a proportional effort–abundance relationship?* Only the models with untransformed effort covariates (M3 and M5).
2. *Was a proportional relationship better estimated by an offset than by a covariate when there was collinearity among covariates?* No, there was no evidence of this given the moderate collinearity in this simulation – provided that all collinear variables were included in the model (Fig. 2b). Across a gradient of collinearity, the covariate’s effort estimate stayed unbiased with near-nominal coverage, but its variance increased with collinearity (Fig. S3); the offset term avoids this uncertainty when proportionality holds. The issues of collinearity and parameter estimation are well discussed elsewhere and apply equally here (Zuur et al. 2010; Dormann et al. 2013).
3. *Was an effort smoother affected by the misspecification of the non-linear but correlated temperature covariate?* Yes, a small amount. This is seen comparing M4 and M6 for D4 and D8. When temperature was correctly specified (M6) the model seemed to better recover the shape of the effort–abundance relationship (Fig. 2), but the change was small.
4. *Were there any limitations to using an effort smoother?* There was some overfitting at the edges due to fewer data and less certainty estimating the expected value (Fig. 2), but otherwise no. For scenarios with fewer observations, or high model complexity, having additional parameters and increased estimation uncertainty could be considered a limitation.
5. *Are there limitations to an offset term?* Only when there is non-proportionality in the effort–abundance relationship, which when unmodelled can also affect the accuracy of other estimates (e.g. poor recovery of temperature for M1 in D4).

### Recommendations

Based on this analysis and other studies, I make the following recommendations and guidance for the modelling of sampling effort and the use of offset terms:

- Including effort as an offset +offset(log(effort) or covariate + log(effort) are suitable for modelling a proportional effort–abundance relationship, provided that the effort covariate is log-transformed (for a log-link GLM). Only the offset guarantees proportionality so is preferred when proportionality is likely, because it avoids bias due to measurement error or model misspecification, and can have better accuracy amidst standard noise (Table 3). Effort variables likely to be proportional would be the area or duration surveyed by observational methods.
- If non-proportionality is possible (e.g. through the saturation of sampling gear) effort should be included as a covariate, because this can model a proportional relationship plus deviations from it. Using a smoother + s(log(effort)) term is most useful due to its flexibility and robust shrinkage; the log-transformation of effort is important even for smoothers (Table S2).
- Consider constraining the wiggliness of the effort smoother to avoid unlikely relationships, e.g. using parameter ‘k’: +s(log(effort), k = 4), and additional shape constraints if necessary (Pya and Wood 2015). Using the variable selection arguments bs=”ts” or 'select' (Wood 2017) are only useful if effort is potentially unimportant.
- Using an effort covariate is an obvious choice for including effort in both parts of a delta (hurdle) model, as offsets have a different use in binomial models and are typically only used in the positive component (Thorson 2019; but see Shelton et al. 2014), although there are related alternatives to delta models (Thorson 2018). However, the effort–probability relationship for a binomial model has less theoretical basis and cannot be proportional. Adding an untransformed effort variable (to a logit-link model) assumes that *absolute* changes in effort have a consistent effect on the log-odds of sampling, which may be more realistic than a log-transformed effort variable (Fig. S4). The only defensible offset structure is likely of log-transformed effort with a ‘clog-log’ link function (e.g. Mannocci et al. 2020) which approximates a proportional effort–detection probability relationship at small probabilities.
- Machine learning is sometimes used as a GLM alternative, and in these cases effort is included as a covariate or the response changed to *Y*/*effort* (Leathwick et al. 2006; Li et al. 2015; Smith et al. 2020). Given the lack of a link function, log-transforming the covariate is no longer essential but is probably still useful, especially for methods like Bayesian additive regression trees which have more constraints on their fitted responses (Chipman et al. 2010) compared to methods like random forests.
- Understanding the data is essential: consider whether your measure of effort is likely to be proportional based on controlled experiments or expert advice, e.g. a doubling of trawl area can double abundance, but a doubling of underwater viewing distance of a camera probably does not (Smith et al. 2017) and should be converted to viewing volume before inclusion as an offset term.
- Also evaluate aspects such as interactions between effort and other variables, e.g. imagine captures of small mammals or fish are highest at certain times of day (e.g. dawn, dusk, or overnight), but live-traps (or nets) may be left for longer periods for practical reasons; the effort–abundance relationship would vary across a ‘duration’ variable. A smoother covariate on duration, or a categorical effort covariate (checked within 6 h vs. left 6 h+) would be worth exploring. Evaluate cautiously a plot of effort versus abundance to evaluate the relationship as it is likely conditional on other effects.
- Multiple offsets can be used if there is more than one effort dimension (e.g. Kortello et al. 2024) but this is the same as using their product: *Y*/(*effort*_1_×*effort*_2_). Calculating this product can evaluate whether this model structure is logical. Otherwise use either: one offset term and additional variables as covariates, or all terms as covariates (Maunder and Punt 2004). Carefully note which covariates are assumed multiplicative and which proportional (Grüss et al. 2019). When using effort products, consider whether (for example) 10 camera traps deployed for 5 nights are as effective as 5 camera traps deployed for 10 nights (both equal 50 camera-trap-nights); spatial and temporal replication may not be interchangeable in this way (Kays et al. 2020). If the two effort components are not exchangeable they should be modelled separately as covariates.
- Like with any covariates, very strong collinearity will inflate the variance/uncertainty of parameter estimates, so remove such covariates when inference of the effort effect is important. Including effort as an offset versus a covariate does somewhat delineate an interest in standardisation (an offset) or inference (covariate).
- Although I encourage prior consideration of whether effort should be a covariate or offset, a simple statistical test can identify support from the data (requires a log link). If *p* < 0.05 (or better yet 0.01 or 0.005) the effort covariate is supported (i.e. *β*_2_ ≠ 1 in Eq. 2):

model_covariate<-glm(Y~X+log(Effort)).

model_offset<-glm(Y~X+offset(log(Effort))).

anova(model_covariate,model_offset,test=“Chisq”).

### Additional considerations

Additional points to consider when modelling effort include:

- Effort can be an *endogenous variable*, and this can affect how effort is modelled. Imagine that survey effort increases in response to animal density, i.e. when more animals are observed or caught, observers exert more sampling effort. This can certainly occur in fisheries (i.e. fishing effort increase when catches are good; Gaertner and Dreyfus-Leon 2004) and in citizen science surveys (Tang et al. 2021). Including effort as a covariate in this case can confound trends in abundance and effort (see a worked example in Fig. S5). Careful treatment is needed when including effort as a covariate when endogenity is suspected.
- In multi-species models, should all species be considered to have the same effort–abundance relationship? Perhaps, although an effort covariate could be fitted for each species in case of species differences (Smith et al. 2024). An offset may be more robust for rare species for which a covariate may have considerable uncertainty.
- A similar alternative to M7 (Table 1) is modelling abundance per unit effort (*Y*/*effort*) but avoiding pre-model transformation by using a delta model combining binomial and (commonly) gamma components (e.g. Panzeri et al. 2024).
- What do GLMs assume about zeros in the data? Is a zero from (say) 2 h effort equivalent to a zero from 24 h effort? Probably not, and such an occurrence would have no influence on a model with an effort offset term. This would, however, influence an estimated effort covariate. These patterns could also influence a binomial model by including effort as a model ‘weight’ if a covariate is deemed inappropriate.
- Treating effort as a factor is also possible (Grüss et al. 2019), e.g. soak time with four levels (Groeneveld et al. 2003), which could be useful when there are clusters of effort values or insufficient data, and such variables could estimate flexible relationships, with each level estimated separately.

## Supplementary Information

Below is the link to the electronic supplementary material.

Supplementary Material 1

## Data Availability

The simulation code including the simulated data are available at: https://github.com/smithja16/Effort_Offset_Simulation.
