# Supplementary material for: Offset or not: guidance on accounting for sampling effort when modelling abundance data
Source: Oecologia. 2026 Jul 30;208(8):103. doi: 10.1007/s00442-026-05944-z (PMC13423905; doi:10.1007/s00442-026-05944-z)
Supplement: Supplementary file 1 — Supplementary Material 1 [file 442_2026_5944_MOESM1_ESM.pdf]

Supplementary Material

Offset or not: guidance on accounting for sampling effort when modelling abundance data

James A. Smith[1,2*]

[1] Port Stephens Fisheries Institute, New South Wales Department of Primary Industries, Locked Bag 1, Nelson Bay, NSW 2315, Australia

[2] School of Biological, Earth and Environmental Science, University of New South Wales, Sydney, NSW, 2052, Australia

[*] james.a.smith@dpi.nsw.gov.au

Table S1. Raw mean absolute error (MAE) values from the repeated cross validation process, for the six models and eight data scenarios. Values should only be compared within data scenarios.

	**M1**	**M2**	**M3**	**M4**	**M5**	**M6**
**D1**	44.39	44.38	44.44	44.38	44.45	44.38
**D2**	45.71	45.65	45.93	45.64	45.63	45.64
**D3**	44.01	43.15	43.31	42.79	42.78	42.81
**D4**	43.44	42.95	43.23	42.38	42.37	42.4
**D5**	15.4	15.44	15.56	15.38	15.39	14.91
**D6**	44.22	44.25	44.67	44.25	44.34	44.27
**D7**	4.61	4.39	4.39	4.39	4.39	4.39
**D8**	13.37	13.23	13.4	13.1	13.1	12.65

Table S2. Delta-AIC values for all models and data scenarios. For each row, delta-AIC measures the increase in AIC from the model with the lowest AIC (= 0). Delta-AIC values ≤ 2 are highlighted grey, because these can be considered equivalent in terms of parsimony.

	**M1**	**M2**	**M3**	**M4**	**M5**	**M6**
**D1**	0	1.4	4.5	1.4	4.5	1.4
**D2**	0	1.2	9.8	1.2	2.9	1.2
**D3**	42.7	25.0	37.6	1.6	0	1.6
**D4**	29.4	19.4	35.1	0.9	0	0.9
**D5**	82.9	84.6	102	82.0	82.6	0
**D6**	0	2.0	22.9	2.0	10.5	2.0
**D7**	124	0	0	0	0	0
**D8**	141	130	166	91.5	94.0	0

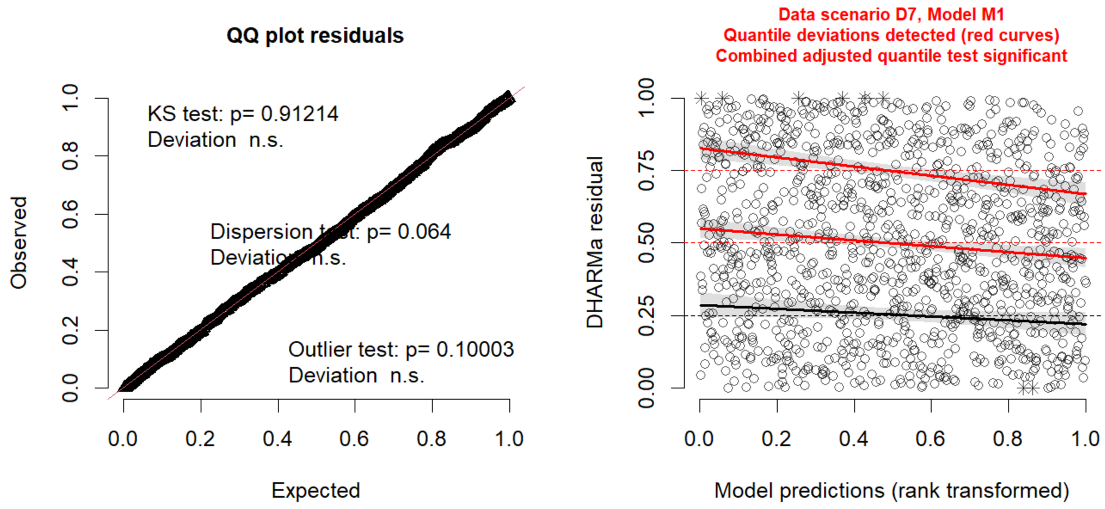

Fig. S1a. DHARMa residuals plot for scenario D7 and model M1.

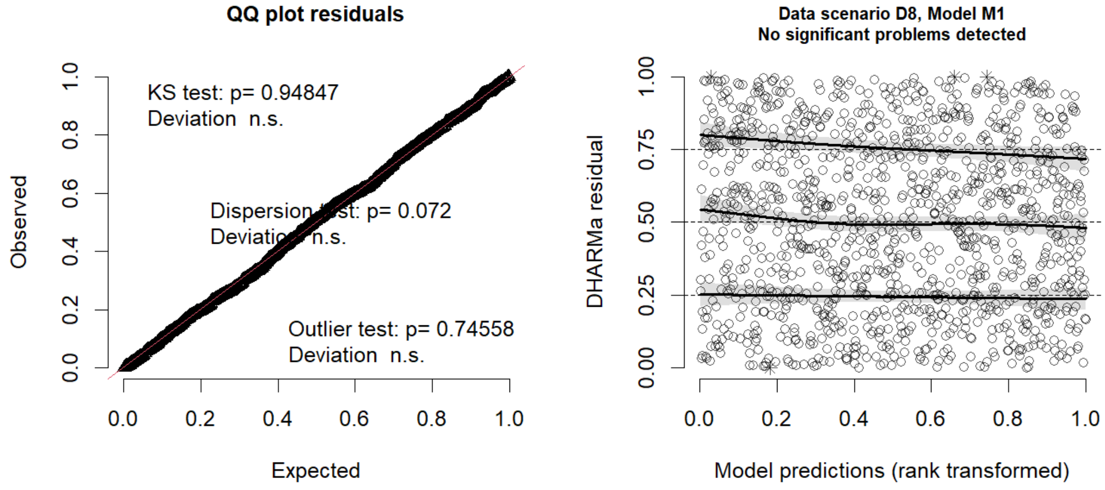

Fig. S1b. DHARMa residuals plot for scenario D8 and model M1.

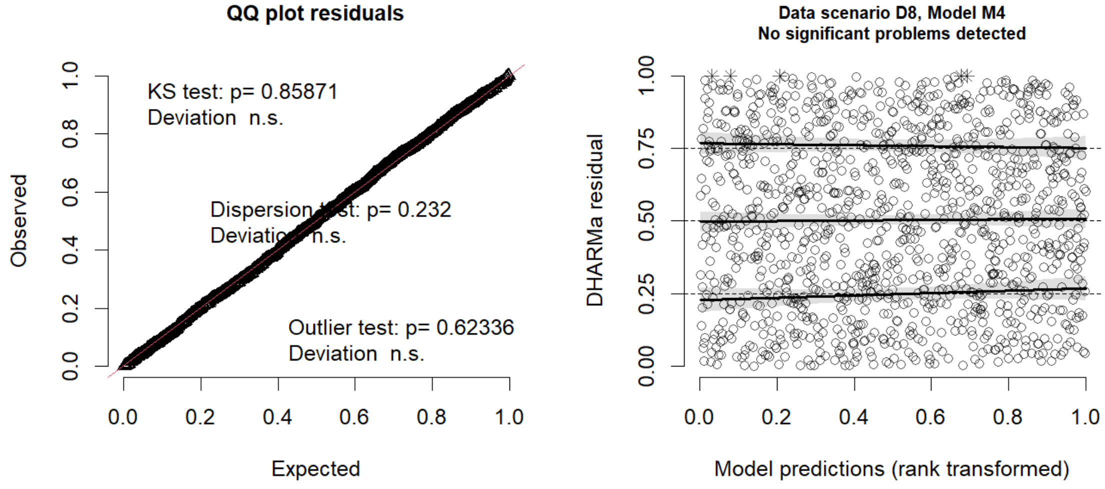

Fig. S1c. DHARMa residuals plot for scenario D8 and model M4.

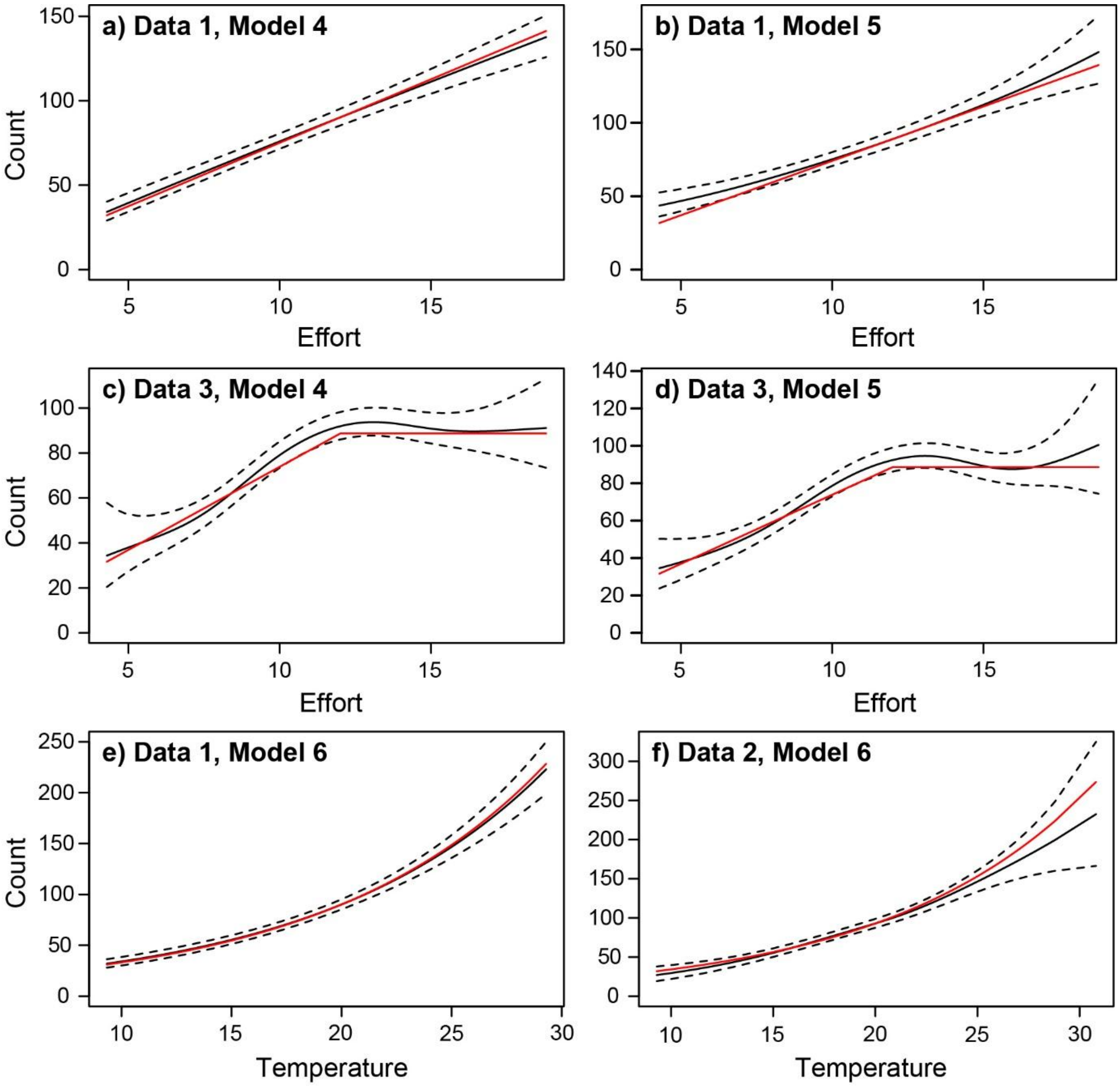

Fig. S2. Examples of how the success of effort and temperature smoothers were evaluated. In a-d) if the true effort–abundance relationship (red line) was within the 95% confidence interval for the estimated smoother (black lines), then the model was deemed successful at recovering the true effort–abundance relationship. In e-f) the same process was used but for the ‘linear’ temperature–abundance relationship.

Table S3. Summary table of parameter recovery for relevant models and data types; T = temperature, Int = intercept, SB = Site B, SC = Site C. Green cells indicate when the 95% confidence interval of an estimate encompassed the true value used to generate the data. Three results which reflect unexpected poor recovery of parameters are highlighted purple.

	Model 1				Model 2					Model 3					Model 4					Model 5					Model 6				
	T	Int	SB	SC	T	Int	SB	SC	E[1]	T	Int	SB	SC	E	T	Int	SB	SC	E[3]	T	Int	SB	SC	E[5]	T[4]	Int	SB	SC	E[5]
D1														[2]															
D2														[2]															
D3									[7]					[7]															
D4									[7]					[7]															
D5	[6]				[6]					[6]				[2]	[6]					[6]					[5]				
D6														[2]															
D7																													
D8	[6]				[6]				[7]	[6]				[7]	[6]					[6]					[5]				

[1] Parameter recovery for the 'log(effort)' variable in M2 was considered successful when the 95% interval for the slope included 1, or 0 (D7)

[2] It is not possible for the 'effort' variable in M3 to recover the correct parameter which is on the log scale, except for a slope = 0 for D7

[3] Parameter recovery for the 's(log(effort))' variable in M4-M6 was considered successful for the proportional data scenarios (D1, D2, D5, D6) when a slope=1 (or slope=0 for D7) linear-predictor effort term was within the confidence interval for the smoother for the extent of effort values; likewise for the threshold data scenarios (D3, D4, D8; Fig. S1)

[4] Parameter recovery for the 's(Temperature)' variable in M6 was considered successful for when a slope=0.1 linear-predictor temperature term was within the confidence interval for the smoother for the extent of temperature values (Fig. S1); when the temperature effect was non-linear (D5, D8) success was evaluated by eye (see [5])

[5] The temperature smoother for D5 was accurately symmetrical and correctly centred on T_{mean} = 20; the smoother for D8 was less symmetrical and centered on 18

[6] It was expected that linear temperature terms could not recover a domed effect (D5 and D8)

[7] It was expected that the linear effort terms could not recover the threshold effort relationship (D3, D4, D8)

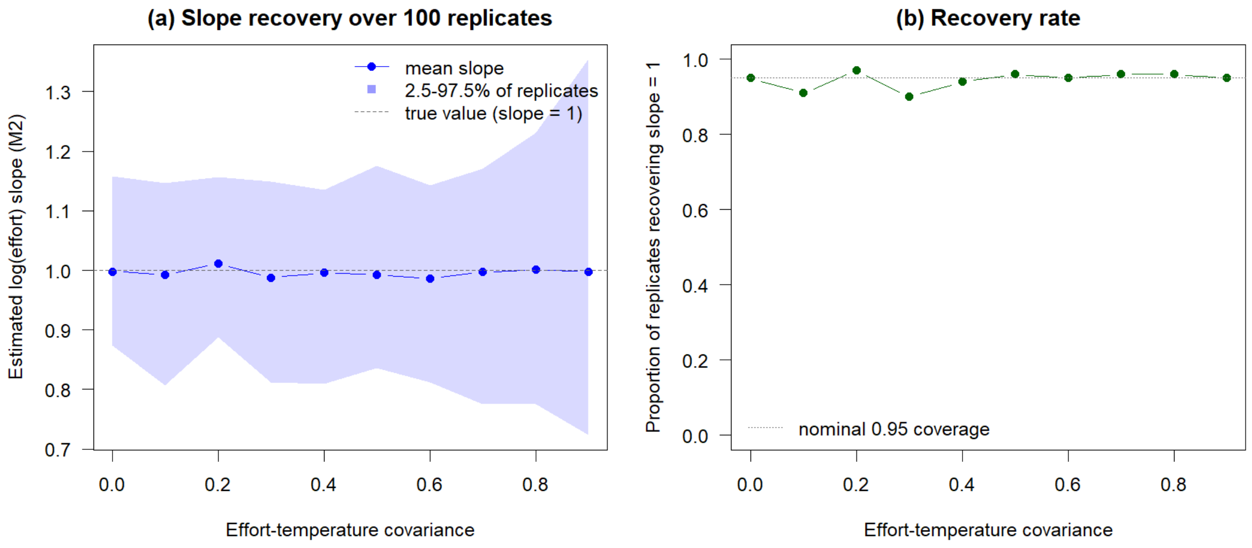

Fig. S3. Sensitivity of the estimated effort effect to the strength of collinearity. For each level of effort-temperature covariance (0 to 0.9 in steps of 0.1), 100 independent datasets were simulated (1,200 negative-binomial counts each, dispersion $\theta = 3$) under a proportional effort-abundance relationship with a linear temperature effect, with collinearity induced only between effort and temperature (as per scenario D2). Two models were fit to every dataset, M1 (effort as offset model) and M2 (effort as covariate model). The figure illustrates the estimated effort coefficient from M2. a) Estimated log(effort) slope from M2: the blue points and line show the mean across the 100 replicates, the shaded band shows the 2.5–97.5% range of the replicate estimates, and the dashed line marks the true value of one (the slope that the offset M1 assumes). b) Recovery rate: the proportion of the 100 replicates whose 95% confidence interval for the M2 slope contained the true value of one; the dotted line marks the nominal 0.95 coverage. The mean slope stays centred on one at every level of collinearity (no bias), while the spread of estimates widens as collinearity increases (the central 95% range roughly doubles from covariance 0 to 0.9), and coverage remains close to the nominal 0.95 throughout (indicating that the standard errors correctly track the growing uncertainty). Collinearity therefore inflates the uncertainty of the estimated effort effect without biasing it. The choice between an offset and a covariate is unaffected by collinearity in this simulation, and where proportionality holds the offset avoids this added uncertainty by not estimating the effort coefficient.

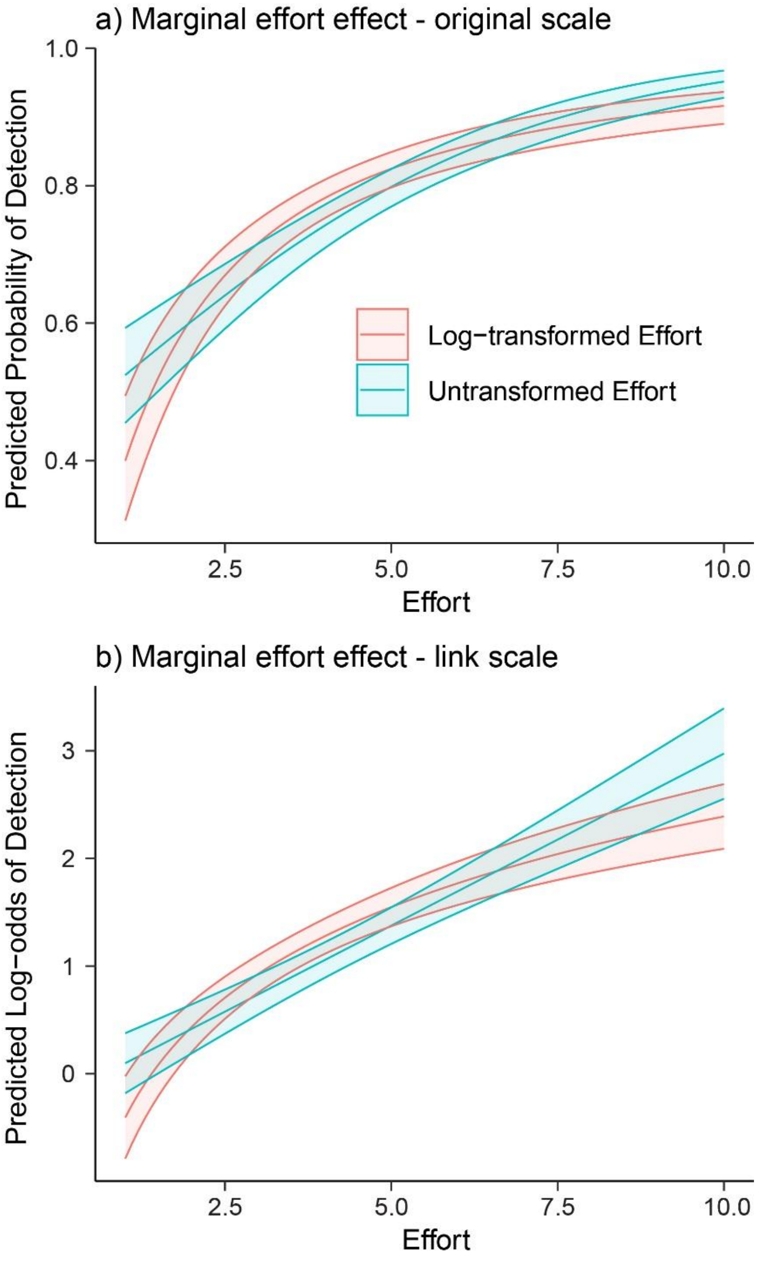

Fig. S4. The difference in the assumed effort-probability relationship when effort is included as a log-transformed (red) or untransformed (blue) covariate in a binomial GLM with logit-link. The untransformed covariate is linear with log-odds (b, link scale), whereas the log-transformed covariate is non-linear on both scales. These are simulated data, and code for generating this figure is shared in the GitHub repository.

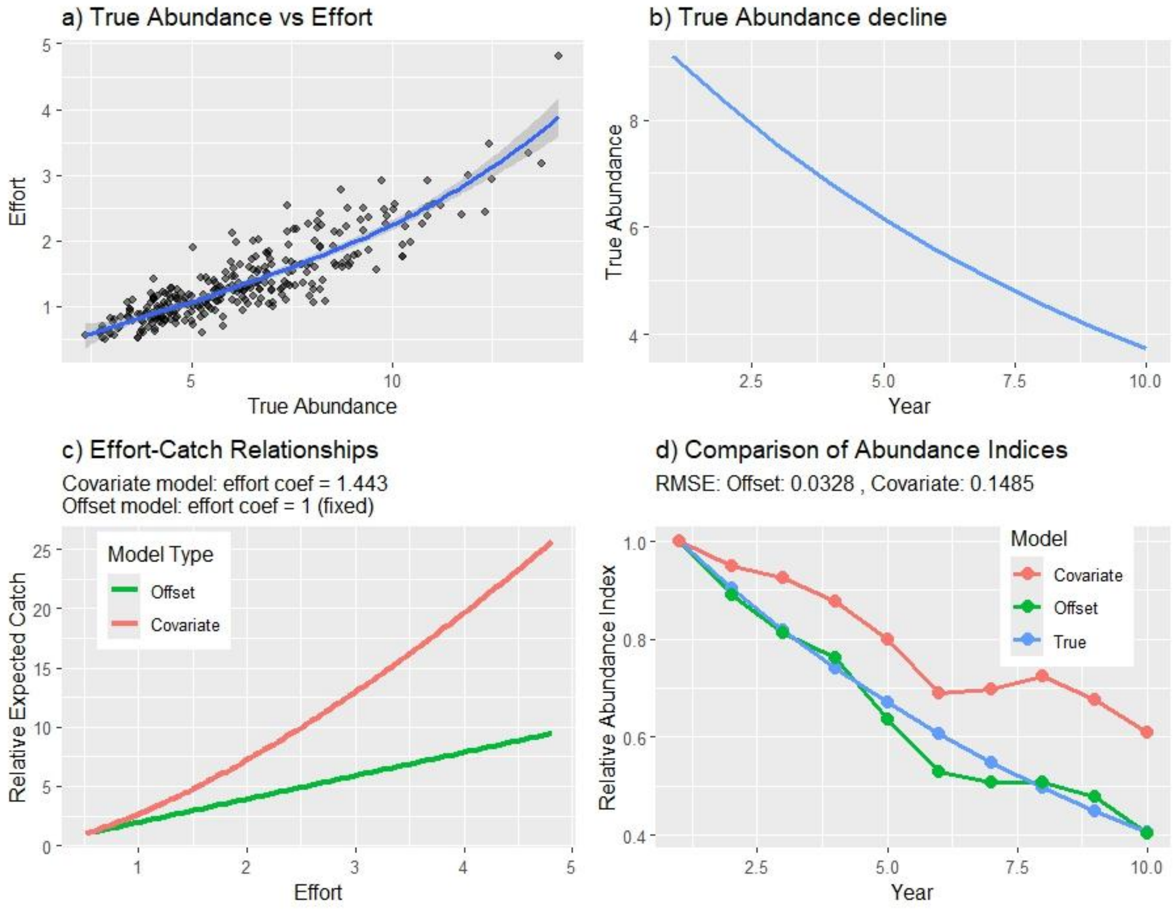

Fig. S5. In this fishery-related scenario, effort is proportional to abundance (i.e. you double the effort you double the abundance). But we also see that 1) when fish abundance is higher, catch rates are higher, 2) when catch rates are higher, fishers respond by increasing effort; this creates a positive correlation between effort and abundance (a). If there is a trend in abundance, in this case declining (b), it's possible to conflate these two patterns (a, b) with a non-proportional effort-abundance relationship. In this case, a model including effort as a covariate incorrectly attributes some of the year-to-year decline in abundance to the effort variable, whereas the model with the offset correctly forces proportionality (c). If we use these models in a catch rate standardization, the covariate model consistently overestimates abundance relative to the true trend (d). The code for this simulation is shared in the GitHub respository.
